# The causal pathway effects of a physical activity intervention on adiposity in children: The KISS Study cluster randomized clinical trial

**DOI:** 10.1111/sms.13741

**Published:** 2020-06-22

**Authors:** Rodrigo Antunes Lima, Lars Bo Andersen, Fernanda Cunha Soares, Susi Kriemler

**Affiliations:** ^1^ Institute of Sports Science University of Graz Graz Austria; ^2^ Research Group on Lifestyles and Health University of Pernambuco Recife Brazil; ^3^ Department of Sports Medicine Norwegian School of Sport Sciences Oslo Norway; ^4^ Faculty of Education, Arts and Sport Western Norway University of Applied Sciences Bergen Norway; ^5^ Epidemiology, Biostatistics and Prevention Institute University of Zürich Zürich Switzerland

**Keywords:** children, exercise, metabolism, obesity, physiology

## Abstract

**Background:**

Very little information on the potential mechanisms of the physical activity interventions effects on adiposity is available. We evaluated the possible mediating factors of a physical activity school‐based intervention on the sum of skinfolds in children.

**Methods:**

This is a cluster randomized trial, secondary analysis of the KISS study. Children (n = 499) from the first and fifth grades were randomly assigned to intervention or control group. Adiposity was estimated by four skinfolds, aerobic fitness assessed by the shuttle run test, and insulin, triglycerides, total cholesterol, high‐density lipoprotein (HDL), and glucose collected via fasting blood samples.

**Results:**

The intervention affected aerobic fitness (0.140 SD, 95% CI 0.011 to 0.270), triglycerides (0.217 SD, 95% CI −0.409 to −0.025), cholesterol/HDL ratio (−0.191 SD, 95% CI −0.334 to −0.047), glucose (−0.330 SD, 95% CI −0.538 to −0.121), and skinfolds (−0.122 SD, 95% CI −0.189 to −0.056). No intervention effect on insulin was found. We observed that changes in aerobic fitness impacted children's triglycerides and cholesterol/HDL ratio and consecutively the glucose levels mediating 30% of the intervention effect on skinfolds.

**Conclusions:**

Our findings provided evidence of the positive metabolic distress caused by a physical activity intervention on adiposity levels in children.

## 1 INTRODUCTION

The increasing rates and the health‐related consequences of overweight and obesity during childhood^1^ have stimulated researchers on conducting interventions targeting adiposity in children.^2^ Although small randomized controlled studies have demonstrated the beneficial effects of physical activity programs on adiposity in children,^3^ large epidemiological school‐based interventions have mostly failed in changing adiposity in children.^4^, ^5^, ^6^, ^7^ Nevertheless, the World Health Organization considers schools to be an ideal setting for promoting physical activity in children and targeting overweight and obesity.^8^

Analyzing well‐successful school‐based interventions might support the development of improved and more comprehensive protocols to be tested in other settings, cultures, and in larger scales.^9^, ^10^ A school‐based study conducted in Switzerland reported positive effects of a physical activity program on several health outcomes in children, including the effects on sum of skinfolds.^11^ Kriemler et al^11^ reported differences between groups by 0.12 *z*‐score units, which corresponded to an approximate 2 mm (or 6% in relative values) of the children's mean sum of skinfolds at baseline. Although the results reported are encouraging, further aspects can still be investigated.

It is not clear which mechanisms mediated the intervention effects on adiposity in the study conducted by Kriemler et al.^11^ Examining the factors that mediate physical activity interventions effects on adiposity will enable researchers to understand which mechanisms are related to intervention effects on adiposity in children and support the design of more successful interventions. Very little information on the potential mechanisms of the physical activity interventions effects on adiposity is available.^12^ To the best of our knowledge, no study has evaluated the mechanistic pathway between physical activity intervention and adiposity in a school‐based study conducted with children. The randomized design in the study conducted by Kriemler et al^11^ reduces potential confounding, supporting a more appropriate analysis of the possible mediating factors accounting for the intervention effects on the outcome.^13^ Besides intervention effects on adiposity, Kriemler et al^11^ reported intervention effects on aerobic fitness, triglycerides, HDL cholesterol, and glucose levels^11^; thus, it is possible to evaluate whether any of those parameters mediated the intervention effects on adiposity.^13^ Therefore, we evaluated the possible mediating factors of a physical activity school‐based intervention on the sum of skinfolds in the same group of children evaluated by Kriemler et al.^11^

## 2 MATERIAL AND METHODS

### 2.1 Design and study population

This is a secondary analysis of the cluster randomized trial: “Effect of school based physical activity programme (KISS study).” The study took place in two of the 26 provinces of Switzerland (Aargau and Baselland), comprising about 10% of the Swiss population. Recruitment started in autumn 2004, and the actual study took place between August 2005 and July 2006. The design of the study has been previously described in detail.^14^ Supplementary material presents a summarized description of the sample selection and randomization, and Supplementary Figure S1, reproduced from Zahner et al 2006,^14^ details the flowchart of participants. All participating children and their parents gave informed consent. The study was approved by the ethics committees of the University of Basel and the ETH of Zürich, as well as by the Cantonal Ethical Committee of Aargau, Switzerland (159/04). Trial registration Current Controlled Trials: ISRCTN15360785.

### 2.2 Intervention

The intervention was targeted at both the cluster and the individual levels and was based on a socio‐ecological conceptual model focusing on increasing daily physical activity.^11^, ^14^, ^15^ Briefly, children in both groups had three physical education lessons each week, which are compulsory by law. The intervention group had two additional physical education lessons on the remaining school days. A team of expert physical education teachers prepared all five physical education lessons for the children in the intervention group. All intervention classes received the same curriculum. The three compulsory weekly physical education lessons (45 minutes each) were given by the usual classroom teachers according to the specified curriculum, whereas the two additional weekly lessons (45 minutes each) were taught mostly outdoors by physical education teachers. In addition, three to five short activity breaks (2‐5 minutes each) during academic lessons—comprising motor skill tasks such as jumping or balancing on one leg, power games, or coordinative tasks—were introduced every day. The children received daily physical activity homework of about 10 minutes' duration prepared by the physical education teachers. This included aerobic, strength, or motor skill tasks such as brushing their teeth while standing on one leg, hopping up and down the stairs, rope jumping, or comparable activities. Children and parents in the control group were not informed about the existence of the intervention program in other schools. The teachers in the control group knew about the intervention arm but were not informed about its content. No incentives for participating in the study were offered to the children.

### 2.3 Measurements

Baseline (August 2005) and follow‐up (June 2006) measurements took place at school within the same 3‐week period for all children; the intervention period lasted 9 months. All assessors were trained in a pilot study 2 months before the main study. Assessors responsible for the measurements were blinded to the group allocation for all measurements except skinfold and waist circumference measures. School laws prescribed that the last two measurements were made by designated physicians who knew the group allocation.

In this study, the primary outcome was the sum of four skinfolds, whereas glucose, cholesterol and HDL ratio (cholesterol/HDL ratio), triglycerides, insulin, and aerobic fitness were tested as mediators of the intervention effect on the sum of skinfolds. Skinfold thickness was measured in triplicate to the nearest 0.5 mm with Harpenden calipers (HSK‐BI, British Indicators). We calculated the sum of four sites (triceps, biceps, subscapular, and suprailiacal).^16^ Blood was drawn in the morning, while the child was fasting for measurement of glucose and lipids as previously described.^14^ We used the 20‐m shuttle run test to determine aerobic fitness.^17^ Ten children did the test together, but each child had a researcher assigned who was checking adequate test procedures. Table 1 describes the number of children in the intervention and control groups with complete data in each variable before and after the intervention.

**Table 1:** Number of children with complete data in the control and intervention groups before and after the intervention

Health parameters	Control		Intervention	
	Before	After	Before	After
Skinfolds	185	206	284	293
Glucose	97	100	234	198
Chol/HDL ratio	109	120	242	238
Triglycerides	99	109	236	216
Insulin	97	100	234	199
Shuttle run	187	190	285	270

Note

Chol/HDL ratio is the ratio between total cholesterol and HDL‐cholesterol

### 2.4 Statistical analyses

We used Stata 16 for windows (StataCorp LP). Descriptive analyses are shown in means and standard deviations. For the main analysis, we *z*‐transformed the variables for each time point (baseline and post‐intervention) by grade, sex, and intervention group‐specific means and standard deviations. Although Kriemler et al^11^ already reported intervention effects on the primary outcome and on the mediators, except for insulin, we decided to rerun the analysis evaluating the intervention effects on adiposity and on each possible mediator before estimating the factors that mediated the intervention effects on adiposity, which is the primary aim of this investigation. All the analyses were conducted as follows.

Structural equation modeling was used to evaluate the effects of the intervention on each of the primary outcomes and on the mediators. We used the maximum likelihood for missing values, which does not exclude a participant in the analysis because of a missing value in one of the variables. Thus, we avoided selection bias in our analysis. Our proposed model is shown in Supplementary Figure S2. All mediators that were influenced by the intervention remained in the model to estimate the indirect effect of the intervention on the sum of skinfolds. Direct effect refers to the direct relationship between two variables. Indirect effects refer to the relationship between two variables that is mediated by a third variable or a group of variables on the pathway, and total effect is the sum of the direct and indirect effects. More details on the final diagram that estimated the indirect effects of the intervention on the sum of skinfolds are presented in the Results section. The diagram will be considered with a good fit to the data if the following parameters are meet: Comparative Fit Index (CFI)> 0.95, and the root mean square error of approximation (RMSEA) <0.08.^18^

## 3 RESULTS

Among the 499 children who had complete data and were included in the main analysis, 226 were enrolled in the first grade (mean age of 6.89 ± 0.32) and 273 in the fifth grade (mean age of 11.12 ± 0.58). Children from intervention (n = 293) and control groups (n = 206) did not differ regarding their age (*P* = .71). In the control group, 51.5% were girls, whereas in the intervention group, 52.6% of girls were included (*P* = .81). Moreover, children with a baseline assessment but no follow‐up assessment did not differ from the remaining children in terms of age, sex, and the variables at baseline (data not shown).

Table 2 presents descriptive information on aerobic fitness, insulin, triglycerides, cholesterol/HDL ratio, glucose, and the sum of skinfolds at baseline and follow‐up by intervention group. It also reports the effect of the intervention in each variable of interest. In summary, the intervention affected the sum of skinfolds and all the mediators with the exception of insulin (*P* = .878).

**Table 2:** Health parameters in children according to group

Health parameters	Control		Intervention		Adjusted difference at follow‐up^a^	
	Before	After	Before	After	Std. β	(95% CI)
Aerobic fitness (stages)	5.81 (2.05)	6.69 (1.89)	5.65 (2.26)	6.83 (2.17)	0.140	(0.011 to 0.270)
Insulin (mmol/L)	7.48 (3.42)	8.03 (5.84)	7.06 (3.83)	7.33 (3.94)	−0.016	(−0.216 to 0.185)
Triglycerides (mmol/L)	0.64 (0.31)	0.67 (0.31)	0.59 (0.24)	0.60 (0.25)	−0.217	(−0.409 to −0.025)
Chol/HDL ratio	2.52 (0.51)	2.47 (0.51)	2.63 (0.58)	2.46 (0.56)	−0.191	(−0.334 to −0.047)
Glucose (mmol/L)	4.60 (0.45)	4.71 (0.38)	4.56 (0.39)	4.58 (0.34)	−0.330	(−0.538 to −0.121)
Skinfolds (mm)	31.88 (14.08)	34.89 (18.98)	32.25 (13.32)	32.19 (14.54)	−0.122	(−0.189 to −0.056)

Note

Values are means and standard deviations (SD) unless stated otherwise.

^a^ Adjusted difference, in standard deviations, between intervention and control groups in relation to each health parameter; analysis adjusted for sex, school class, and the respective health parameter at baseline. Chol/HDL ratio is the ratio between total cholesterol and HDL‐cholesterol.

**Figure 1:**
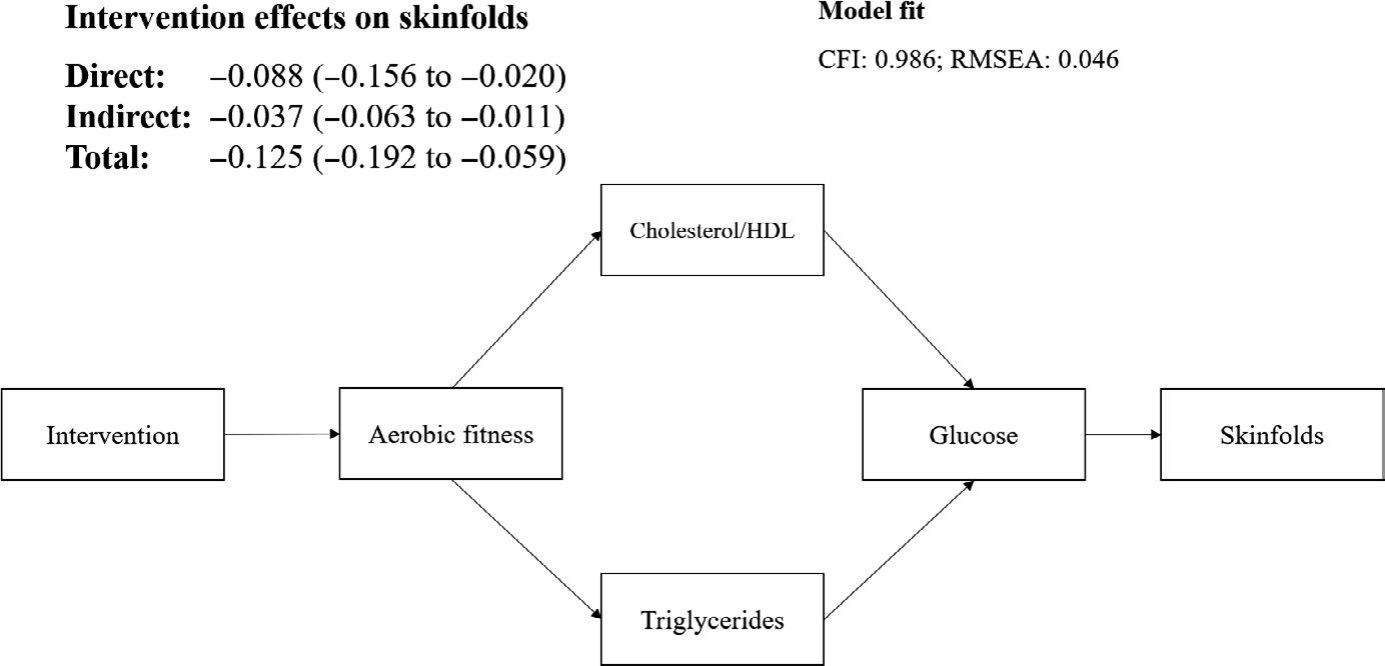
Standardized path coefficients of the direct, indirect, and total effects of the intervention on the sum of skinfolds. CFI refers to the Comparative Fit Index; RMSEA refers to the root mean square error of approximation. Adjusted for sex, school class, and the respective outcome at baseline

Since insulin was not affected by the intervention, the diagram shown in Supplementary Figure S2 for analyzing the mediation effects of the intervention on the sum of skinfolds was modified. Figure 1 shows the final diagram used for the mediation analysis and the direct, indirect, and the total effects of the intervention on the sum of skinfolds. Changes in aerobic fitness, triglycerides, cholesterol/HDL ratio, and glucose levels mediated 30% of the intervention effects on skinfolds. More specifically, our diagram suggests that the intervention increased the aerobic fitness, which affected children's triglycerides and cholesterol/HDL ratio and consecutively the glucose levels. The combination of those changes resulted in lower sum of skinfolds in the intervention group compared to the control group by 0.037 SD (−0.063 to −0.011) (indirect effect of the intervention). Our model presented good fit to the data: CFI: 0.986 and RMSEA: 0.046 (Figure 1).

## 4 DISCUSSION

As demonstrated previously,^11^ the intervention affected the sum of skinfolds of the children attending the intervention schools. Moreover, our proposed framework observed that 30% of the intervention effect on skinfolds was mediated via changes in aerobic fitness, triglycerides, cholesterol/HDL ratio, and glucose levels. Our framework highlights the importance of higher physical activity intensities in targeting adiposity in children, since increases in aerobic fitness led to changes in metabolic parameters culminating with reduced adiposity. To the best of our knowledge, this is the first study to disentangle the biological pathways that affected adiposity in a large‐scale epidemiological study with children.

There are few physiological mechanisms that can explain the indirect effects of the intervention on adiposity in the current study.

### 4.1 Intervention effect on the aerobic capacity

Increased mitochondrial density might have had a twofold impact on the intervention effects in our study.^19^ Firstly, in combination to the enhanced heart capacity and blood flow stimulated by the maintenance of physical activity level, higher mitochondrial density diminishes the metabolic stress to homeostasis caused by each bout of physical activity, which in turn increases the efficacy in energy utilization and the aerobic capacity.^20^ Those changes might explain the effects of the intervention on aerobic fitness in our study. Secondly, physical activity has been shown to induce mitochondrial biogenesis in the adipose tissue,^21^, ^22^ increasing the metabolic demand in the adipose tissue caused by the intervention.^20^

### 4.2 Intervention effects on triglycerides and glucose levels

Adenosine triphosphate (ATP) synthesis might underlie some of the other metabolic changes related to the effects of the intervention in adiposity observed in our study. Since intramuscular ATP is relatively small, prolonged physical activity stimulates ATP resynthesizes. In physical activities lasting less than 1 minute, ATP resynthesizes occurs mainly by the substrate‐level phosphorylation via the breakdown of creatine phosphate and during the conversion of glucose units, derived essentially from breakdown of intramuscular glycogen to lactate.^23^, ^24^ Additional substrates are essential to maintain skeletal muscle metabolism during prolonged physical activity.^25^, ^26^ Initially from glycogenolysis and subsequently from gluconeogenesis, the liver releases glucose into the circulation.^12^ In addition, in the adipocyte, the hydrolysis of its triglycerides is increased, releasing long‐chain non‐esterified fatty acids into the bloodstream.^12^ This chain of events might be related to the indirect effects of the intervention on adiposity via triglycerides and glucose levels.

### 4.3 Intervention effects on lipids and cholesterol

The indirect impact of the intervention on adiposity via HDL/cholesterol might be due to the positive relationship between aerobic capacity and lipid profile regulation. This relationship is associated with the enzymatic modulation of lecithin‐cholesterol acyltransferase (L‐CAT), which is involved in the esterification of cholesterol.^27^ In addition, cholesteryl ester transfer protein (CETP) is one of the facilitators of the transport of cholesterol ester particles of HDL‐c to other lipoproteins,^28^, ^29^ since both L‐CAT and CETP are part of the mechanism in which the excess of cholesterol is removed from the peripheral tissues and both pathways are stimulated by physical activity.^27^ Moreover, aerobic exercise has been shown to activate the hydrolysis of the triacylglycerols (TAG) from very low‐density lipoprotein (VLDL) by lipoprotein lipase (LPL), which partly results, in the production of beneficial HDL‐c particles.^30^, ^31^ LPL is situated on the inside of capillaries and is closely associated with aerobic fitness.^19^

### 4.4 The non‐significant intervention effects on insulin

The intervention did not affect fasting insulin levels in this sample of children, although previous investigations have reported effects of physical activity interventions on insulin and on insulin resistance.^32^, ^33^ Physical activity is believed to increase microvascular recruitment, enhancing glucose delivery to the cells.^34^ In addition, physical activity stimulates the GLUT4‐mediated glucose transport, which can be an additional pathway for increasing insulin sensibility caused by higher physical activity level. A study conducted with type 2 diabetic and obese participants observed that physical exercise led to increased expression and function of several proteins involved in insulin‐signal transduction, specially the insulin receptor substrates 1 and 2 (IRS‐1 and IRS‐2).^35^ It is possible that the statistically non‐significant impact of the intervention on insulin is due to a measurement bias. Insulin is very sensitive to even a few non‐fasted individuals within a group.^36^, ^37^ Even when all are fasting the variation is high.^36^, ^37^ Moreover, insulin depends on the physical activity level of preceding days^37^, ^38^; acutely, physical activity empties glycogen from the muscle cell,^39^, ^40^ which could also partly explain the non‐significant results.

### 4.5 Strengths and limitations

The representativeness of the sample and the relatively simple intervention protocol are probably the biggest strengths of this study, increasing the probability of reproducing the findings in other cultures. Although this is the first large school‐based study to estimate the indirect effects of a physical activity intervention on adiposity in children, our model explained only 30% of the intervention effects. Thus, around 70% of the effects were due to changes in other factors not measured in this study or measurement error. We encourage future studies to investigate other markers that might underlie the impact of a physical activity intervention on adiposity in children. A better understanding of the physiological pathways will support the design of more effective interventions targeting adiposity in children.

This large school‐based physical activity intervention effectively targeted adiposity in children, and importantly, changes in aerobic fitness, triglycerides, cholesterol/HDL ratio, and glucose mediated 30% of the intervention effects. Our findings provided evidence of the positive metabolic distress caused by a physical activity intervention, which resulted in lower levels of adiposity in children exposed to the intervention.

## 5 PERSPECTIVE

Our findings support the premise that physical activity impacts adiposity in children via changes in aerobic fitness, triglycerides, cholesterol/HDL ratio, and glucose levels. This biological pathway accounted for 30.0% of the intervention effects on adiposity, demonstrating a physiological distress responsible for decreased adiposity in children. Moreover, our findings suggest that higher physical activity intensities might be preferable to target adiposity in children, since increases in aerobic fitness were in the beginning of the chain of relationships leading to a reduction in adiposity. Future studies should test the possible mediation role of insulin in the impact of a physical activity intervention on adiposity in children despite the non‐significant result reported in the current investigation. Since there is a reasonable body of evidence suggesting the importance of insulin sensitivity in the physical activity‐adiposity relationship^33^. Moreover, there are other biological pathways accounting for the reduction in adiposity in children that we did not account in this study. Deeper understanding on the biological causal effect related to adiposity loss is pivotal to target childhood obesity and should be evaluated in future studies.

## CONFLICT OF INTERESTS

None of the authors declares a conflict of interest.

## AUTHOR CONTRIBUTIONS

RL and LA designed this study. RL and FS conducted the statistical analysis. All authors contributed to the interpretation of the results and drafted the manuscript. SK made substantial contribution to the conception of the KISS study and acquisition of data. All authors revised the manuscript and contributed to the content. All authors approved the final manuscript as submitted and agree to be accountable for all aspects of the work.

## Supporting information

Fig S1

Fig S2

Supplementary Material
