# Supplementary figures and images for: The causal pathway effects of a physical activity intervention on adiposity in children: The KISS Study cluster randomized clinical trial
Source: Scand J Med Sci Sports. 2020 Jun 22;30(9):1685–91. doi: 10.1111/sms.13741 (PMC7496602; doi:10.1111/sms.13741)

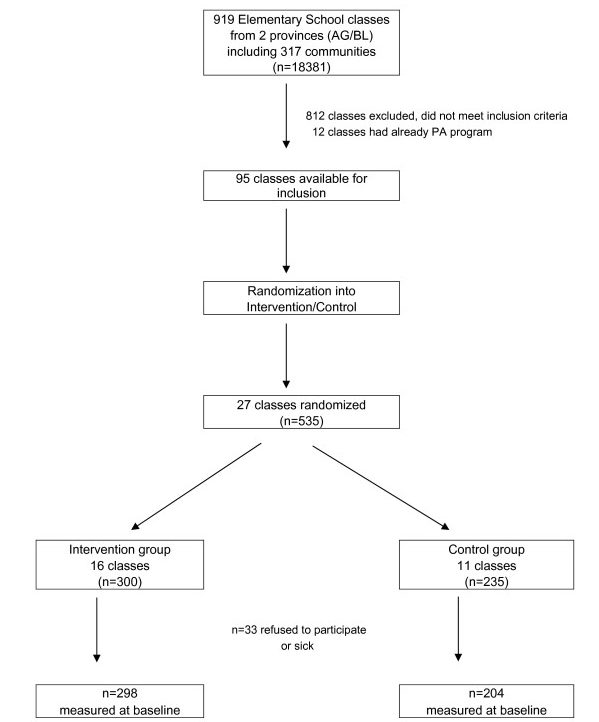

Supplement: Supplementary file 1 — Fig S1 [file SMS-30-1685-s001.tif]

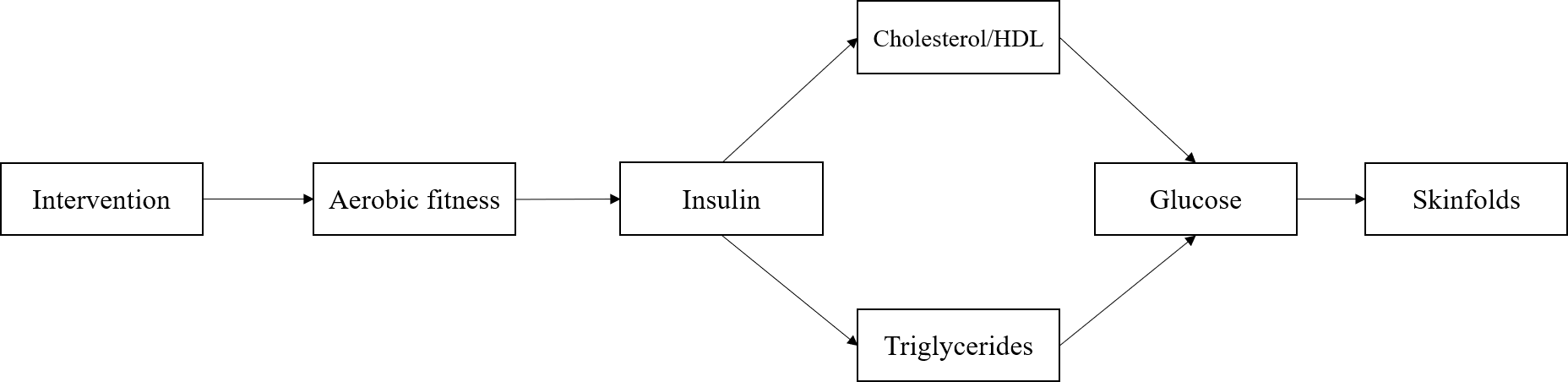

Supplement: Supplementary file 2 — Fig S2 [file SMS-30-1685-s002.tif]
