# Supplementary material for: The causal pathway effects of a physical activity intervention on adiposity in children: The KISS Study cluster randomized clinical trial
Source: Scand J Med Sci Sports. 2020 Jun 22;30(9):1685–91. doi: 10.1111/sms.13741 (PMC7496602; doi:10.1111/sms.13741)
Supplement: Supplementary file 3 — Supplementary Material [file SMS-30-1685-s003.docx]

Supplementary Material

In summary, 28 of 190 of the consenting classes were selected on the basis of a computer‐generated random number table that was in the hands of a person not involved in the study. Participating schools fulfilled our eligibility criteria: rural or urban localization, a prevalence of 10%‐30% migrants as in the Swiss population, and, for practical reasons, the presence of at least a first grade and a fifth‐grade class in each school. Intervention and control schools were located in provinces that were comparable as regards socioeconomic status of the population and recreational facilities at school. In the intervention arm, 16 classes from nine schools were selected (eight classes in the first grade and eight classes in the fifth grade). In the control arm, 12 classes from six schools were selected (six classes in the first grade and six classes in the fifth grade). Note that, in the protocol,^1^ 11 classes not 12 should have been selected. To avoid contamination of treatments, the randomization occurred in the school level. However, the school director had no role during the whole study period, and all parts of the intervention were done at the class level. Classes from the intervention and control groups were located in different villages or towns.

Reference

1. Zahner L, Puder JJ, Roth R, et al. A school‐based physical activity program to improve health and fitness in children aged 6–13 years (“Kinder‐Sportstudie KISS”): study design of a randomized controlled trial [ISRCTN15360785]. BMC Public Health. 2006;6:147.
